# Two decades of gastric and gastroesophageal junction cancer surgery

**DOI:** 10.1007/s00432-023-04719-w

**Published:** 2023-03-31

**Authors:** Patrick S. Plum, Aylin Pamuk, Atakan G. Barutcu, Christoph Mallmann, Emanuel Niesen, Felix Berlth, Thomas Zander, Seung-Hun Chon, Stefan P. Moenig, Alexander Quaas, Christiane J. Bruns, Arnulf H. Hoelscher, Hakan Alakus

**Affiliations:** 1 Department of General, Visceral, Cancer and Transplantation Surgery, University Hospital Cologne, Kerpener Str. 62, 50937 Cologne, Germany; 2 Department of Visceral, Transplant, Thoracic and Vascular Surgery, University Hospital Leipzig, Leipzig, Germany; 3 Department I of Internal Medicine, Center for Integrated Oncology (CIO), University Hospital Cologne, Cologne, Germany; 4 Gastrointestinal Cancer Group Cologne (GCGC), Cologne, Germany; 5 Department of General, Visceral and Transplant Surgery, University Medical Center, Mainz, Germany; 6 Service de Chirurgie Viscéral, Hôpitaux Universitaires de Genève, Geneva, Switzerland; 7 Institute of Pathology, University Hospital Cologne, Cologne, Germany; 8 Center for Esophageal Diseases, Elisabeth-Krankenhaus, Essen, Germany

**Keywords:** Gastric carcinoma, Gastroesophageal junction adenocarcinoma, MAGIC trial, Chemotherapy, Multimodal treatment

## Abstract

**Purpose:**

Diagnosis and treatment of gastric and gastroesophageal junction cancer have undergone many critical changes during the last two decades. We addressed the question of how clinical reality outside of clinical trials has changed for gastric and gastroesophageal junction cancer patients in a European center for upper gastrointestinal surgery.

**Methods:**

In this retrospective cohort study, patients undergoing (sub)total gastrectomy for gastric or gastroesophageal junction adenocarcinoma between 1996 and 2017 in a tertiary upper gastrointestinal center were included. The time was divided into a) before (1996–2006) (pre-CTx) and b) after (2006–2017) (CTx) the MAGIC trial. Data were comprehensively analyzed for demographics, tumor stage, perioperative treatment, surgery, histopathology, and survival rates (SR).

**Results:**

737 patients (32% female) underwent gastrectomy, 255 patients in the pre-CTx era and 482 patients in the CTx era. The median age was 65 years and the median follow-up was 27.5 months for surviving patients. Around 16.9% of patients received neoadjuvant treatment in the pre-CTx era versus 46.3% in the CTx era. The 3-year survival rate (3-YSR) was 46.4% in the pre-CTx and 60.9% in the CTx era (*p* < 0.001). For pretreated patients, 3-YSR was 39.0% (pre-CTx) versus 55.3% (CTx) (*p* = 0.168). Survival rate (SR) for locally advanced tumor stages (cT3/cT4) was higher when neoadjuvant therapy was administered (3-YSR: 56.7% vs 40.6%; p = 0.022). There were no significant differences according to sex (*p* = 0.357), age (*p* = 0.379), pT category (*p* = 0.817), pN stage (*p* = 0.074), cM stage (*p* = 0.112), Laurén classification (*p* = 0.158), and SRs (3-YSR: 60.3% vs 59.4%; *p* = 0.898) between the MAGIC and FLOT regimens.

**Conclusions:**

Survival rates have dramatically improved for gastric cancer patients during the last two decades. MAGIC and FLOT regimens showed similar results in the postsurgical follow-up.

## Introduction

Gastric cancer incidence is continuously decreasing worldwide, but it remains the fourth most common cancer disease in males (683,754) and fifth in females (349,947) (Bray et al. 2018).

Fortunately, the diagnosis and treatment of gastric and gastroesophageal junction (GEJ) cancer have undergone many important improvements during the last years. In 2006, the MAGIC (Medical Research Council Adjuvant Gastric Infusional Chemotherapy) phase-III trial was a major landmark for the treatment of gastric and gastroesophageal junction cancer patients. It showed a 5-year survival rate (5-YSR) of 36% for patients treated with a perioperative regimen of epirubicin, cisplatin, and 5-fluorouracil (ECF) compared to 23% for patients treated with surgery alone (Cunningham et al. 2006). Similar results were achieved by the French ACCORD07-FFCD 9703 study (Ychou et al. 2011), leading to the recommendation favoring multimodal perioperative treatment for cT3 and cT4 tumors in current gastric cancer guidelines (S3-Leitlinie Magenkarzinom. 2019). Recently, the ECF regimen has been replaced by the perioperative fluorouracil, leucovorin, oxaliplatin, and docetaxel (FLOT) regimen, enhancing chances of achieving pathological complete regression by 10% and an increase in estimated 5-YSR by 9% (Al-Batran et al. 2019, 2016).

Despite standardized surgical treatment (systematic D2 lymphadenectomy (LAD) with the goal of complete resection (R0)), prognosis remains poor with a 5-YSR of 30%-35% due to high recurrence rates, lymphogenic micro-metastasis and distant metastasis (Barnes et al. 2016; Chon et al. 2017).

In the present study, we addressed the question of *how* clinical reality outside of clinical trials has changed in detail during the last two decades. We conducted a comprehensive analysis of different therapeutic approaches in the treatment of gastric cancer, which were carried out at the University Hospital Cologne over two decades. A special focus was set on relevant survival benefits for patients treated in the CTx era and prognostic factors associated with improved outcomes.

We performed the study in a Western high-volume center for upper gastrointestinal surgery covering every single patient of the recent two decades thoroughly.

## Methods

### Patients

Between 01.05.1996 and 31.05.2017, consecutive patients which underwent gastrectomy at the University Hospital Cologne for histologically proven adenocarcinoma of the stomach or gastroesophageal junction (GEJ) were documented in a prospectively established database (Chief of Surgery: CJ Bruns since 01.05.2016, AH Hoelscher 01.05.1996-30.04.2016). The period was divided into two eras based on the operation date of the first patient receiving epirubicin-containing chemotherapy according to the MAGIC trial: (a) 1996–2006 defined as the pre-CTx era and (b) 2006-2017 defined as the CTx era. The date of censoring for the patients’ follow-up was 11/19/2019.

Data on patient demographics, diagnostic procedures including gastroscopy and endoscopic ultrasound, previous operations, therapeutic procedures including chemotherapy regimen and surgical treatment, disease progression, and complications were collected from the discharge reports. Pathological data, including grading and response to neoadjuvant therapy, was obtained from the original histopathological report. Depth of tumor infiltration (T), lymphonodular invasion (N), presence of distant metastasis (M), and margin status (R) were evaluated according to the 7^th^ edition of the TNM International Union Against Cancer (UICC) classification. Furthermore, chemotherapeutic procedures were extracted from the tumor board review reports. Follow-up data were collected from the in-house aftercare and readmission reports. These data analysis was performed according to the criteria of the local ethics committee of the University Hospital of Cologne (application number: 19-1480).

### Staging and treatment

Tumor staging was performed by computed tomography, gastroscopy, endoscopic ultrasound, and in some cases laparoscopy before neoadjuvant therapy or surgery. Patients treated with neoadjuvant therapy were restaged to evaluate therapy response and to rule out inoperability before surgery. Restaging was performed via clinical examination, contrast CT of the thorax and abdomen and gastroscopy (without further pathological biopsies) knowing that certain phenomena, such as the occurrence of micrometastases with extranodal lymphatic metastasis or so-called tumor budding, cannot be detected with this method. Chemotherapeutic groups were identified as follows: (i) epirubicin-containing chemotherapy (MAGIC) (ii) taxane-containing chemotherapy (FLOT) (iii) a combination based on thymidylate synthase inhibitor (5-FU or capecitabine) and platin (PLF) (iv) radio-chemotherapy (RCTx) and (v) other chemotherapy regimens (remnant groups). Resection was performed as total or subtotal gastrectomy with or without transhiatal extension and either with D2 LAD or a reduced (< D2) or an extended version (D2 +) of it, depending on tumor size, site, histological subtype according to Laurén and local spread. Reconstruction of the gastrointestinal passage was conducted following the current clinic guidelines (Roux-en-Y ± pouch, Billroth II, or colon interposition) at this time. Postoperative complications were reviewed in medical records and reclassified according to Clavien-Dindo.

### Data management and statistical analyses

Patients were divided into two groups for analysis. The first analysis group comprised pre- and CTx era subgroups. The second analysis compared chemotherapy followed by surgery versus stand-alone surgery subgroups. The statistical analyses were performed with the SPSS 25.0 software (IBM Inc., Armonk, NY). For the analyses of categorical variables, the Chi-square statistic and for continuous variables, the Student’s t-test was used. The hazard ratio for treatment alone and adjusted for baseline stratification factors were analyzed by a cox-proportional-hazards model. Subgroup analyses were performed to examine the treatment effect for various baseline characteristics using a cox-regression model (multivariate analysis, MVA). A two-sided p-value of under 0.05 was considered significant. Kaplan–Meier survival plots were calculated for overall survival (OS) and compared using the log-rank test and 3-/5-year survival rates (YSR). Data on patients with no event or loss to follow-up were censored at the last seen date. Overall survival was defined as the time from the date of operation to death**.**

## Results

### Patient characteristics

For this database, 941 Caucasian patients which underwent gastrectomy between May 1996 and May 2017 were retrospectively examined. After excluding patients without adenocarcinoma of the stomach or the GEJ, 737 patients were included in the present study (67.7% male, 32.3% female, median age: 65 years). Of these, 255 (34.6%) underwent gastrectomy in the pre- and 482 (65.4%) in the CTx group. Both groups had similar demographic data and distribution of tumor location (*p* = 0.234) as seen in Table 1.

**Table 1:** Characteristics of all patients and grouped after pre- and post-MAGIC era

		Total (*n* = 737)	pre-MAGIC-era (*n* = 255)	post-MAGIC-era (*n* = 482)	*p*-value
Age	Median	65 years	67 years	65 years	0.249
Age distribution	11–20 years	1	0	1	0.452
	21–30 years	4	0	4	
	31–40 years	28	10	18	
	41–50 years	75	28	47	
	51–60 years	155	43	112	
	61–70 years	214	80	134	
	71–80 years	220	81	139	
	81–90 years	39	14	25	
	91–100 years	1	0	1	
Sex	Male	499 (67.7%)	172 (67.5%)	327 (67.8%)	0.914
	Female	238 (32.3%)	83 (32.5%)	155 (32.2%)	
Tumor site	GEJ	267 (36.3%)	89 (34.9%)	178 (37.1%)	0.234
	Proximal gastric	60 (8.2%)	26 (10,2%)	34 (7.1%)	
	Midbody (corpus)	193 (2.3%)	56 ( 22%)	137 (28.5%)	
	Distal gastric	162 (22%)	64 (25.1%)	98 (20.4%)	
	Whole gastric	19 (2.6%)	7 (2.7%)	12 (2.5%)	
	Gastric stump and Anastomosis	34 (4.6%)	13 (5.1%)	21 (4.4%)	
cM	cM0	538 (83.8%)	167 (80.3%)	371 (85.5%)	0.095
	cM1	104 (16.2%)	41 (19.7%)	63 (14.5%)	
Pretreatment	Primary operation	433 (58.8%)	212 (83.1%)	221 (45.9%)	< 0.001
	Neoadjuvant treatment	304 (41.2%)	43 (16.9%)	261 (54.1%)	
Extent of resection	transhiatal extented Gastrectomy	300 (40.7%)	111 (43.5%)	189 (39.2%)	0.121
	Total gastrectomy	318 (43.1%)	95 (37.3%)	223 (46.3%)	
	Total gastrectomy with Subtotal esophagectomy	9 (1.2%)	2 (0.8%)	7 (1.5%)	
	Subtotal gastrectomy	70 (9.5%)	31 (12.2%)	39 (8.1%)	
	Gastrectomy of the remnant gastric	38 (5.2%)	15 (5.9%)	23 (4.8%)	
	FUNDUS resection	2 (0.3%)	1 (0.4%)	1 (0.2%)	
Reconstruction	Roux-en-Y with pouch	100 (13.8%)	31 (12.4%)	69 (14.6%)	0.001
	Roux-en-Y without pouch	600 ( 83%)	206 (82.4%)	394 (83.3%)	
	Billroth 2	9 (1.2%)	0.095)	0	
	Colon interposition	10 (1.4%)	3 (1.2%)	7 (1.5%)	
	other	4 (0.6%)	1 (0.4%)	3 (0.6%)	
Lymphadenectomy	< D2	20 (2.8%)	12 (5.1%)	8 (1.7%)	0.010
	D2	458 (65.2%)	160 (67.8%)	298 (63.9%)	
	> D2	224 (31.9%)	64 (27.1%)	160 (34.3%)	
(y)pT	(y)pT0-T1	162 (22.6%)	43 (17.6%)	119 (25.2%)	0.054
	(y)pT2	86 (12%)	28 (11.5%)	58 (12.3%)	
	(y)pT3-T4	469 (65.4%)	173 (70.9%)	296 (62.6%)	
(y)pN	(y)pN0	319 (43.8%)	100 (39.7%)	219 (46%)	0.107
	(y)pN1	115 (15.8%)	36 (14.3%)	79 (16.6%)	
	(y)pN2	101 (13.9%)	36 (14.3%)	65 (13.7%)	
	(y)pN3	193 (26.5%)	80 (31.7%)	113 (23.7%)	
Margin status	R0	652 (90.6%)	227 (90.4%)	425 (90.6%)	1.0
	R1-R2	68 (9.4%)	24 (9.6%)	44 (9.4%)	
Laurén-classification	Intestinal	175 (39.1%)	93 (39.1%)	82 (39%)	0.358
	Diffuse	232 (51.8%)	119 (50%)	113 (53.8%)	
	Mixed	41 (9.2%)	26 (10.9%)	15 (7.1%)	
Dindo-Clavien- Classification	0	353 (50.8%)	117 (50.2%)	236 (51.1%)	0.070
	1	80 (11.5%)	32 (13.7%)	48 (10.4%)	
	2	100 (14.4%)	41 (17.6%)	59 (12.8%)	
	3a	66 (9.5%)	18 (7.7%)	48 (10.4%)	
	3b	65 (9.4%)	15 (6.4%)	50 (10.8%)	
	4a	9 (1.3%)	2 (0.9%)	7 (1.5%)	
	4b	4 (0.6%)	0	4 (0.9%)	
	5	18 (2.6%)	8 (3.4%)	10 (2.2%)	
Hospital stay	Mean	18.95 days	21.23 days	17.81 days	0.005
Survival	3-YSR	54.9%	46.4%	60.9%	< 0.001
	5-YSR	47.6%	38.8%	55.1%	

*GEJ* Gastroesophageal junction, *YSR* year survival rate

### Treatment

#### Surgery

The characteristics of the surgical procedures are summarized in Table 1. The extent of surgical resection did not significantly differ between pre- and CTx groups (*p* = 0.121).

In the pre-CTx era, D2 level lymphadenectomy (D2 LAD) was performed in 67.8% compared to 63.9% in the CTx era, LAD less than D2 level in 5.1% vs. 1.7% and LAD additional to D2 level in 27.1% vs. 34.3% (*p* = 0.010), respectively. The median number of resected lymph nodes (LN) was 32 LN and similar in both groups (*p* = 0.249).

In total, 96.8% of all reconstructions were done according to Roux-en-Y with or without pouch. In 9 patients (1.2%; with all being in the pre-CTx era) reconstruction according to Billroth II and in 10 patients (1.4%) reconstruction with colon interposition was carried out. There was a statistically significant difference in the distribution of reconstructions according to Billroth II (3.5% in pre- vs 0% in CTx era; *p* < 0.001).

During the entire study period, the rate of tumor-free resection margins (R0) was above 90% (90.6%; 90.4 in pre- vs. 90.6% in CTx era; *p* = 1.0).

Postoperative complications according to the Dindo-Clavien classification were similar for both groups as seen in Table 1 (*p* = 0.070). On average, patients in the CTx group spent 3.4 days less in the hospital (*p* < 0.001).

### Chemotherapy

A total of 304 (41.2%) of the 737 patients received neoadjuvant therapy. Of these, 43 (14.1%) patients received neoadjuvant therapy before the publication of the MAGIC trial and 261 (85.9%) afterward (*p* < 0.001).

118 (38.8%) patients received chemotherapy according to the FLOT regimen, 61 (20.1%) patients received chemotherapy based on the MAGIC trial (ECF), and 30 (9.9%) patients underwent chemotherapy with the PLF regimen, in 6 (2%) patients chemotherapy did not fit into any of the three above-mentioned regimens (remnant group). Forty (13.2%) patients were additionally treated with radiotherapy combined with chemotherapy. More than 87% of the tumors that received radiotherapy were located at the gastroesophageal junction or in the proximal stomach. Here, Radiation was part of the CROSS regimen used for these tumors (Hagen et al. 2012). In 49 (16.1%) patients, the given chemotherapy regimen could not be identified due to insufficient data.

### Pathology

Data on (y)pT and (y)pN stages were available in 717 (97.3%) and 728 (98.8%) cases, respectively. The distribution in the pre- and CTx era is summarized in Table 1.

Histology according to Laurén classification showed no significant difference in distribution between the two operation periods (*p* = 0.358). In both groups, approximately half of the carcinomas were of a diffuse type and about 39% were of intestinal type (see Table 1).

The prevalence of stage (y)pT0-1, (y)pT2, and (y)pT3-4 tumors were 17.6%, 11.5%, and 70.9% among patients of the pre-CTx era versus 25.2%, 12.3%, and 62.6% in CTx patients (*p* = 0.054). Similar results were found considering the (y)pN category: In both subgroups no significant differences considering the (y)pN stage was found (*p* = 0.107) (see Table 1).

In the next step, we focused on the neoadjuvant chemotherapeutic regimen applied to the patients (see Table 2). In the pre-CTx era, patients received exclusively PLF in our cohort, while in the CTx era (ECF and FLOT were predominant). We observed no significant alterations of the ypT stage as well as the ypN stage depending on the chemotherapeutic approach (*p* = 0.883, and *p* = 0.063). The same was true for the occurrence of distant metastases (p = 0.152) or positive resection margins (*p* = 0.347).

**Table 2:** Characteristics of the patients receiving neoadjuvant therapy

		Pretreated (*n* = 304)	PLF (*n* = 30)	ECF (*n* = 61)	FLOT (*n* = 118)	*p*-value
Age	Median	60 years	61 years	58 years	61 years	0.105
Sex	Male	207 (68.1%)	21 (70%)	40 (65.6%)	73 (61.9%)	0.681
	Female	97 (31.9%)	9 (30%)	21 (34.4%)	45 (38.1%)	
Distant metastasis	M0	224 (82.7%)	21 (75%)	48 (90.6%)	93 (80.9%)	0.152
	M1	47 (17.3%)	7 (25%)	5 (9.4%)	22 (19.1%)	
	Missing	33 (10.8%)	2 (6.7%)	8 (13.1%)	3 (2.5%)	
(y)pT	(y)pT0-T1	49 (16.3%)	4 (13.3%)	10 (16.4%)	22( 18.6%)	0.883
	(y)pT2	43 (14.3%)	3 (10%)	10 (16.4%)	15 (12.7%)	
	(y)pT3-T4	208 (69.3%)	23 (76.7%)	41 (67.2%)	81 (68.6%)	
	Missing	4 (1.3%)	0	0	0	
(y)pN	(y)pN0	124 (41.2%)	9 (30%)	29 (47.5%)	46 (39%)	0.063
	(y)pN1	57 (18.9%)	8 (26.7%)	5 (8.2%)	25 (21.2%)	
	(y)pN2	41 (13.6%)	2 (6.7%)	13 (21.3%)	15 (12.7%)	
	(y)pN3	79 (26.2%)	11 (36.7%)	14 (23%)	32 (27.1%)	
	Missing	3 (1.0%)	0	0	0	
Margin status	R0	269 (90.6%)	27 (90%)	53 (88.3%)	109 (94%)	0.347
	R1/R2	28 (9.4%)	3 (10%)	7 (11.7%)	7 (6%)	
	Missing	7 (2.3%)	0	1 (1.6%)	2 (1.7%)	
Histological tumor response	> 50% vital tumor cells	93 (51.4%)	11 (68.8%)	22 (59.5%)	39 (45.9%)	0.404
	50%–10% vital tumor cells	45 (24.9%)	2 (12.5%)	10 (27.0%)	24 (28.2%)	
	< 10% Vital tumor cells	30 (16.6%)	3 (18.8%)	3 (8.1%)	13 (15.3%)	
	Complete response	13 (7.2%)	0	2 (5.4%)	9 (10.6%)	
	Missing	123 (40.5%)	14 (46.7%)	24 (39.3%)	33 (28.0%)	
Survival	3-YSR	51.2%	35.9%	60.3%	59.4%	0.008

*PLF* cisplatin, *5-FU* leucovorin, *ECF* epirubicin, cisplatin, 5-fluorouracil, *FLOT* fluorouracil, leucovorin, oxaliplatin, docetaxel, *YSR* year survival rate

Comparing the MAGIC subgroup with the FLOT subgroup alone, there was no significant difference in the distribution of ypT and ypN stages (*p* = 0.772 and *p* = 0.074) (Table 2). Additionally, there was also no distinction to the PLF subgroup (*p* = 0.883 and *p* = 0.063). In the chi-square test, no significant difference was shown in tumor response between the different chemotherapeutic regimens (*p* = 0.404). Regarding histologic response, data was available for 37 patients in the MAGIC arm. This represents a response rate of 5.4% in 2/37 patients. In the FLOT group, data were available for 85 patients. Thus, 9/85 patients result in 10.6% (*p* = 0.404).

### Outcome

**Fig. 1:**
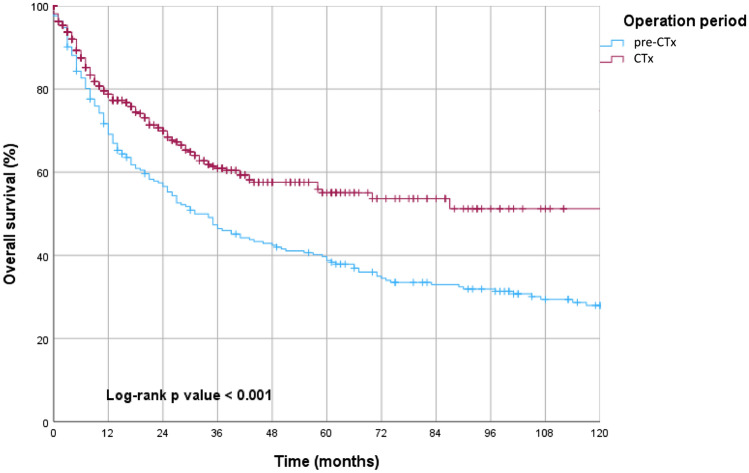
Kaplan–Meier estimates of overall survival in pre-CTx vs. CTx group showing a statistically significant enhancement of overall survival in CTx era

At the time of analysis, the median and mean follow-up for surviving patients were 27.5 and 42 months, respectively. Both 3-year survival (3-YSR) and 5-year survival (5-YSR) improved significantly in the CTx era with 60.9% vs. 46.4% and 55.1% vs. 38.8%, respectively (95% CI 1.267–1.995; *p* < 0.001, Fig. 1).

**Fig. 2:**
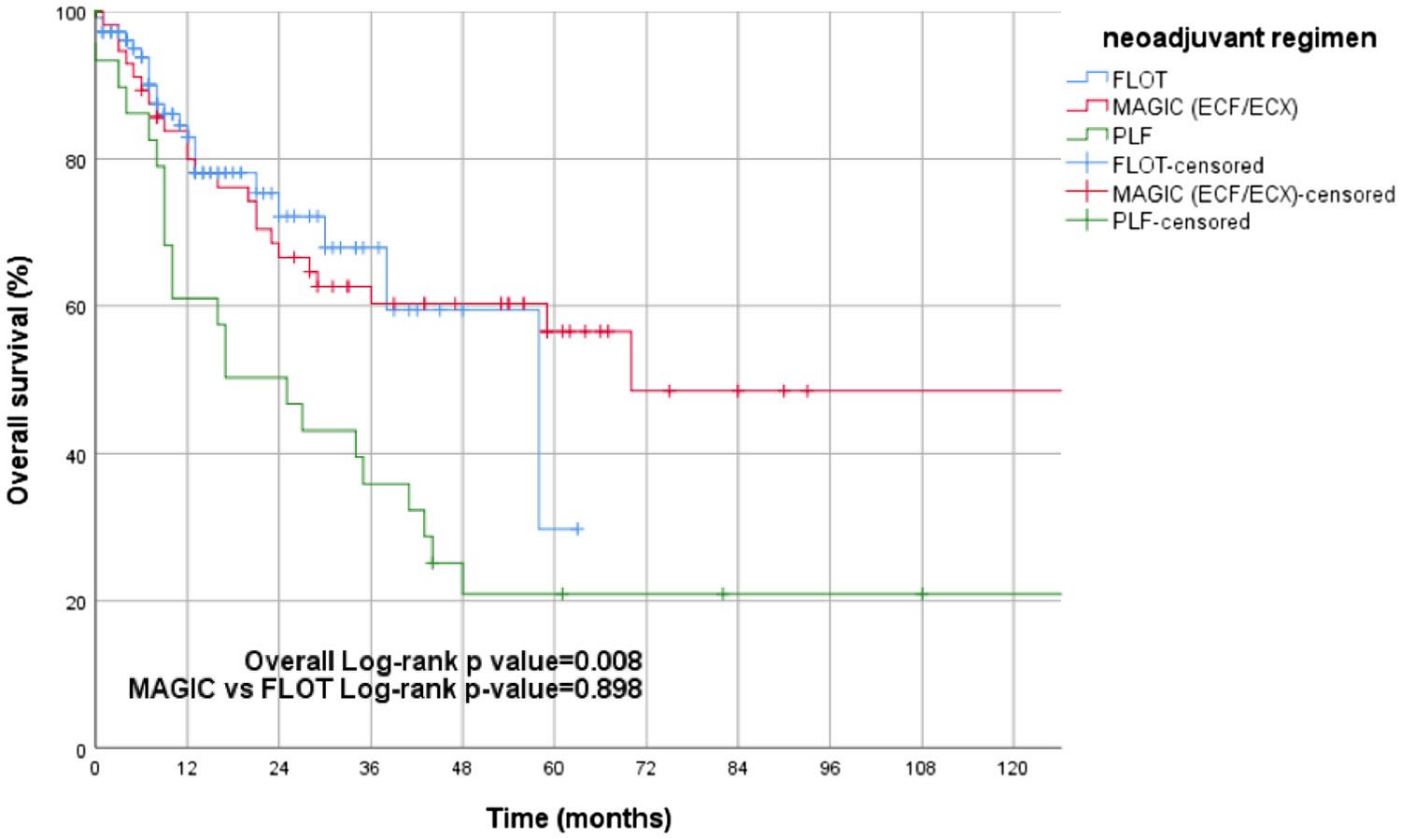
Kaplan–Meier estimates of overall survival in neoadjuvant treatment groups (FLOT vs. MAGIC vs. PLF) showing no significant difference in overall survival between FLOT and MAGIC regimens, whereas patients receiving PLF showcasing poorer survival

The next step was to compare patient outcomes according to the neoadjuvant chemotherapy protocol used. There was no significant distinction in survival rates when comparing patients treated with FLOT or ECF (following the MAGIC regimen) (95% CI 0.519–1.779; *p* = 0.898, Fig. 2). However, in comparisons between the PLF and ECF regimen (95% CI 0.262–0.837; 3-YSR: 35.9% vs 60.3%; *p* = 0.008) and the FLOT regimen (95% CI 0.243–0.836; 3-YSR: 35.9% vs 59.4%; *p* = 0.009), the difference reached significance.

The univariate cox-regression model revealed that the pT stage, pN stage, margin status (R), distant metastasis, histology according to Laurén, tumor location, the extent of resection, the extent of LAD, the type of surgical reconstruction and the operation period (pre-/CTx era) were predictors for overall survival (OS). However, in the multivariate cox-regression analysis, only pT (*p* = 0.005), pN (*p* < 0.001), R status (*p* = 0.008), and cM status (*p* < 0.001) were identified as independent prognostic factors (Table 3).

**Table 3:** Univariate and multivariate analysis of factors associated with prognosis considering the whole study cohort

		Univariate analysis				Multivariate analysis			
		HR	95% CI. for HR		*p*- value	HR	95% CI. for HR		*p*- value
			Lower	Upper			Lower	Upper	
(y)pT	Overall				< 0.001				0.005
	(y)pT0-1	0.2	0.137	0.292	< 0.001	0.445	0.259	0.763	0.003
	(y)pT2	0.409	0.279	0.598	< 0.001	0.560	0.314	0.999	0.050
	(y)pT3-4				Ref				Ref
(y)pN	Overall				< 0.001				< 0.001
	(y)pN0	0.184	0.139	0.244	< 0.001	0.402	0.259	0.622	< 0.001
	(y)pN1	0.284	0.198	0.408	< 0.001	0.393	0.226	0.684	0.001
	(y)pN2	0.488	0.354	0.672	< 0.001	0.630	0.408	0.972	0.037
	(y)pN3				Ref				Ref
Distant metastasis	cM0	0.216	0.161	0.289	< 0.001	0.455	0.301	0.689	< 0.001
	cM1				Ref				Ref
Margin status	R0	0.341	0.245	0.475	< 0.001	0.562	0.368	0.857	0.008
	R1/R2				Ref				Ref
Laurén subtype	Overall				< 0.001				0.289
	Intestinal				Ref				Ref
	Diffuse	1.485	1.123	1.964	0.005	‒	‒	‒	‒
	Mixed	2.555	1.646	3.966	< 0.001	‒	‒	‒	‒
Neoadjuvant therapy	Primary operation	0.869	0.689	1.094	0.232	‒	‒	‒	‒
	Pretretment				Ref	‒	‒	‒	‒
Tumor location	Overall				0.041				0.079
	GEJ				Ref				Ref
	Proximal	1.469	1.009	2.139	0.045	‒	‒	‒	‒
	Corpus	0.743	0.552	1.001	0.051	‒	‒	‒	‒
	Distal	0.959	0.714	1.289	0.783	‒	‒	‒	‒
	Whole gastric	1.286	0.653	2.533	0.466	‒	‒	‒	‒
	Gastric stump and anastomosis	0.949	0.536	1.683	0.859	‒	‒	‒	‒
Extent of resection	Overall				0.001				0.107
	Transhiatal extended gastrectomy				Ref				Ref
	Total gastrectomy	0.756	0.593	0.963	0.024	‒	‒	‒	‒
	Subtotal gastrectomy	0.655	0.435	0.988	0.044	‒	‒	‒	‒
	Fundus resection	0.666	0.093	4.760	0.685				
	Gastrectomy of the remnant stomach	0.859	0.505	1.461	0.575	‒	‒	‒	‒
	Total gastrectomy with subtotal esophagectomy	3.533	1.645	7.589	0.001				
Reconstruction	Overall				0.004				0.083
	Roux-en-Y with pouch				Ref				Ref
	Roux-en-Y without pouch	1.219	0.873	1.702	0.244	‒	‒	‒	‒
	Billroth II	2.186	1.022	4.674	0.044				
	Colon interposition	5.297	1.809	10.208	0.001	‒	‒	‒	‒
LAD	Overall				0.001				0.133
	< D2	2.886	1.631	5.105	< 0.001	‒	‒	‒	‒
	D2	1.092	0.841	1.419	0.507	‒	‒	‒	‒
	> D2				Ref				Ref
Operation period	pre-CTX era	1.590	1.267	1.995	< 0.001				0.160
	post-CTx era				Ref				Ref

*CTx* chemotherapy, *CI* Confidence interval, *HR* Hazard Ratio, *GEJ* Gastroesophageal junction, *LAD* Lymphadenectomy

## Discussion

Despite a declining incidence, gastric cancer remains the third leading cause of cancer deaths worldwide (Bray et al. 2018). In this retrospective study, we investigated how the clinical management of oncologic resection of gastric and gastroesophageal junction cancer has changed over time at a tertiary single center for upper gastrointestinal surgery with an increasing number of patients being referred to our specialized center. With 737 patients included, this analysis is one of the largest gastric cancer studies in Europe. This study details the real-world experience using the knowledge gained in the aforementioned pivotal trials.

It must be noted that this single-center database has several sources of selection bias. Patients with gastric adenocarcinoma not undergoing surgery, caused by advanced tumor stage, high patient age, comorbidities, disease progression under neoadjuvant therapy, and/or patient request were excluded. After all, this database has a retrospective observational design.

Little has changed in the surgical treatment of gastric cancer since 1996. One of the important achievements was gained with the D1D2 Dutch trial. In a multicenter study between 1989 and 1993, 1078 patients with resectable gastric cancer in the Netherlands were randomized into two groups: patients receiving D1 level lymphadenectomy and those receiving D2 level lymphadenectomy. After 15 years of observation, the results showed that D2-LAD was superior to D1-LAD in terms of locoregional recurrence and gastric-cancer-related mortality (Songun et al. 2010).

Although the frequency of D1-LAD has decreased in the CTx era (*p* = 0.010), this cannot be the cause of the improved survival in the CTx era in our cohort, considering the small case numbers of corresponding patients (12 patients (5.1%) vs. 8 patients (1.7%)).

Between both investigated periods, there was no significant difference in tumor location (p = 0.234), in pT/pN-categories, or the cM-status (*p* = 0.054, *p* = 0.107, and *p* = 0.095), in histological type according to Laurén (*p* = 0.358) as well as in the extent of resection (*p* = 0.121). Furthermore, there was no significant difference in this study regarding postoperative complications according to Dindo–Clavien, too. This supports the conclusion that differences in the surgical setting between the operative periods were marginal.

In contrast to surgical therapy, there has been a paradigm shift in the treatment of gastric cancer from surgery alone to multimodal therapy with adjunctive chemotherapy. With the MAGIC trial, a landmark in gastric cancer therapy was set in 2006. Before this trial, several studies failed to demonstrate a survival benefit for neoadjuvant/perioperative chemotherapy in gastric adenocarcinoma (Hartgrink et al. 2004; Janunger and Hafs. 2001).

Following the MAGIC study, the French trial, published in May 2011, compared perioperative chemotherapy with cisplatin and 5-FU (PLF) against primary surgical therapy. In this study, a higher rate of R0 resections and a significant enhancement in disease-free survival and overall survival (24% vs 38%) were shown (Ychou et al. 2011). In light of the above-mentioned studies, perioperative chemotherapy was established as an important treatment option for gastric cancer (S3-Leitlinie Magenkarzinom. 2019).

The 5-YSR of 51.6% after neoadjuvant therapy compared to 34.7% in primarily operated patients is above those reported in the MAGIC trial and the French trial (Cunningham et al. 2006; Ychou et al. 2011). Explanations can be found in the surgical therapy and the tumor characteristics in those studies: patients included in the MAGIC trial received D1 level LAD in 19.5% of cases. As already mentioned, a reduced extent of LAD is associated with poorer survival (Songun et al. 2010). In our cohort, only 2.7% received a D1 level, LAD. In the French trial, 75% of the carcinomas were located in the GEJ or distal esophagus (Ychou et al. 2011). We excluded esophageal carcinomas from our analysis and the proportion of GEJ tumors was only 36.3%. In the literature, tumors located in the proximal stomach are associated with poorer survival than tumors at more distal sites (Petrelli et al. 2017).

Unlike locally advanced gastric cancer, no benefit of neoadjuvant chemotherapy in locally limited gastric cancer (cT2) has been established in the literature. Therefore, the German guidelines from August 2019 only give a weak recommendation (S3-Leitlinie Magenkarzinom. 2019).

Besides investigating the implications of implementation of neoadjuvant therapy in gastric cancer over the course of time, the outcomes after different chemotherapeutic regimens were compared, an aspect which raised growing interest since the publication of the FLOT phase II trial in October 2016 and of the FLOT phase III trial in April 2019. In our study, the FLOT regimen (3-YSR: 59.4%) and the ECF regimen (3-YSR: 60.3%) were superior to the PLF regimen (3-YSR: 35.9%) in terms of OS (*p* = 0.009 and *p* = 0.008, respectively). However, there was no statistically significant difference in OS between the FLOT and MAGIC groups (*p* = 0.898). This result is at odds with those of the FLOT trial (Al-Batran et al. 2019).

In addition, the FLOT4 study demonstrated a significantly higher proportion of patients achieving pathological complete regression for the FLOT regimen (Al-Batran et al. 2019). In the present study, however, no significant difference in the rate of pathological complete response could be demonstrated between the investigated regimens (*p* = 0.404).

In our multivariate analyses (MVA), neoadjuvant therapy was not an independent prognostic factor for survival, even when tested among patients with cT3/cT4 tumors. The reason for this could be the comparatively small number of cases with clinical T-stage information available, considering that neoadjuvant therapy had already been identified as an independent prognostic factor in other studies (Ychou et al. 2011). Despite the lack of significance in the MVA, a survival advantage after neoadjuvant therapy for locally advanced tumors is demonstrated and the improved survival in the CTx era is likely to stem from this collective. Over two-thirds of the patients had locally advanced tumors.

Overall, the present study reflects the evolution of the modern therapeutic concept in the treatment of gastric cancer. The treatment approach has evolved from surgical resection alone to a combination of chemotherapy and surgery. This can also be seen in this monocentric analysis over the course of time. However, this work also has its limitations. Referral patterns have changed over the period. As a large tertiary center for tumors of the upper gastrointestinal tract, patients from all over Germany were increasingly referred to us for surgical treatment in the later study periods, usually after they had already received neoadjuvant chemotherapy from their oncologists close to home according to their preferences. Postoperative follow-up was also performed there. Unfortunately, because there is no central national registry, loss to follow-up was possible. Therefore, it is not possible to identify putative differences in the long-term course beyond this period. In addition, as a result of the retrospective review of the very long time period in this historical cohort, there was a lack of data regarding chemotherapy regimens or other similar issues. As part of the clinical reality and based on the fact that therapeutic concepts have evolved and expanded over time, studies such as the RENAISSANCE (AIO-FLOT5) trial (Al-Batran et al. 2019) have been conducted. This led to the expansion of the indication for surgical resection to include oligometastatic disease. This in turn explains the increase in cM1-positive patients from the pre-CTx to the CTx era.

Since its retrospective character and its limitations mentioned before, a prospective randomized study with a larger case number is needed to clarify the true significance of neoadjuvant therapy in patients with cT2 tumors.

While the FLOT4 trial demonstrated a statistically significant advantage compared to the MAGIC regimen, in the retrospective analysis of our patient collective, we were unable to reproduce this effect and saw no advantage regarding survival when choosing the FLOT regimen over the MAGIC regimen. Although this result seems to be contradicting that of the FLOT4 trial at the first glance, a possible explanation lies in the intention-to-treat design of the FLOT4 trial. In the FLOT arm of the trial, significantly more patients received tumor surgery (336 [94%] *vs* 314 [87%]) (Al-Batran et al. 2019), likely resulting in or contributing to the clear benefit in overall survival. Our analysis only included patients who received surgery, effectively analyzing only the possible difference between the postoperative parts of the respective treatment regimens.

In summary, the current work demonstrates the evolution of multimodal treatment in gastric cancer over the course of time and illustrates the survival benefits from implementation of chemotherapeutic therapy in those patients.

## Data Availability

The datasets generated and/or analyzed during this current study are available from the corresponding author on reasonable request.
